# Navigating fairness aspects of clinical prediction models

**DOI:** 10.1186/s12916-025-04340-3

**Published:** 2025-10-17

**Authors:** Kaustubh Chakradeo, Inchuen Huynh, Sedrah B. Balaganeshan, Ole L. Dollerup, Hjørdis Gade-Jørgensen, Susanne K. Laupstad, Mikkel Malham, Tri-Long Nguyen, Adam Hulman, Tibor V. Varga

**Affiliations:** 1 Department of Public Health, Section for Health Data Science and AI, University of Copenhagen, Copenhagen, Denmark; 2 Department of Public Health, Copenhagen Health Complexity Center, University of Copenhagen, Copenhagen, Denmark; 3 Steno Diabetes Center Aarhus, Aarhus University Hospital, Aarhus, Denmark; 4 Department of Paediatrics and Adolescent Medicine, Copenhagen University Hospital - Amager and Hvidovre, Hvidovre, Denmark; 5 Section of Epidemiology, Department of Public Health, University of Copenhagen, Copenhagen, Denmark; 6 Department of Public Health, Aarhus University, Aarhus, Denmark; 7 Copenhagen, Denmark; 8 Hillerød, Denmark

**Keywords:** Fairness, Algorithms, Algorithmic fairness, Health inequities, Delivery of healthcare, Clinical decision-making, Health personnel, Risk factors, Prediction algorithms, Clinical decision rules

## Abstract

**Background:**

Algorithms are increasingly used in healthcare, yet most algorithms lack thorough evaluation and impact assessment across diverse populations. This absence of comprehensive scrutiny introduces a significant risk of inequitable clinical outcomes, particularly between different demographic and socioeconomic groups.

**Main body:**

Societal biases—rooted in structural inequalities and systemic discrimination—often shape the data used to develop these algorithms. When such biases become embedded into predictive models, algorithms frequently favor privileged populations, further deepening existing inequalities. Without proactive efforts to identify and mitigate these biases, algorithms risk disproportionately harming already marginalized groups, widening the gap between advantaged and disadvantaged patients. Various statistical metrics are available to assess algorithmic fairness, each addressing different dimensions of disparity in predictive performance across population groups. However, understanding and applying these fairness metrics in real-world healthcare settings remains limited. Transparency in both the development and communication of algorithms is essential to building a more equitable healthcare system. Openly addressing fairness concerns fosters trust and accountability, ensuring that fairness considerations become an integral part of algorithm design and implementation rather than an afterthought. Using a participatory approach involving three clinicians and three patients with lived experience of type 2 diabetes, we developed a set of guiding questions to help healthcare professionals assess algorithms critically, challenge existing practices, and stimulate discussions.

**Conclusions:**

We aim to direct healthcare professionals on navigating the complexities of bias in healthcare algorithms by encouraging critical thinking about biases present in society, data, algorithms, and healthcare systems.

**Supplementary Information:**

The online version contains supplementary material available at 10.1186/s12916-025-04340-3.

## Background

The use of algorithms to guide clinical decision-making is rapidly expanding across healthcare. From decision rules like age cut-offs or risk scores for cardiovascular disease (CVD) and type 2 diabetes screening to artificial intelligence (AI)-based models for predicting risks of diabetic retinopathy, these tools aim to *standardize but also personalize* care delivery to improve patient outcomes [1]. However, while some algorithms are extensively evaluated, many are not rigorously tested across diverse patient populations, raising concerns about their real-world impact. This lack of evaluation and the fact that many algorithms ignore key socioeconomic drivers of disease (e.g., income level, education, employment status, housing conditions, and access to healthcare), combined with the increasing reliance on data-driven and AI-based models in healthcare, amplify the risk of sustaining or exacerbating health inequalities [2]. Hence, there is a fundamental need to understand, evaluate, and improve the fairness of algorithms used in healthcare.

Clinical algorithms might perform differently across population strata defined by sensitive attributes like race, ethnicity, sex, or socioeconomic strata [3]. Such disparities may arise due to biological differences or societal, data, and algorithmic biases. Either way, unequal performance can lead to unequal outcomes between privileged and marginalized groups. The past decade has experienced a growing number of assessment tools that identify and mitigate bias in clinical algorithms [4], and reporting on algorithmic fairness has become a mandatory aspect in the development of diagnostic and prognostic clinical prediction models, as emphasized in guidelines such as TRIPOD+AI [5]. Yet, fairness remains a complex and subjective concept, often requiring healthcare professionals to grapple with opaque statistical fairness metrics that quantify disparities in predictive performance [6].

This paper seeks to educate healthcare professionals on navigating this landscape by fostering critical thinking about biases embedded in societies, data, algorithms, and healthcare systems, and introducing key fairness metrics in accessible terms. It also discusses the importance of clear communication about fairness among peers and patients. To contextualize our perspectives as researchers, we took a participatory approach and involved three clinicians and three patients living with type 2 diabetes. To make our article accessible to a broad audience, we included a glossary of technical terms in Table 1.

**Table 1:** Glossary of technical terms

Algorithm	A rule or set of rules aiming to streamline decision-making
Machine learning	A subset of artificial intelligence that involves training algorithms to learn patterns from data, enabling them to make predictions or decisions without being explicitly programmed for each task
Accuracy	Proportion of correct predictions out of all predictions made
False positive	A result that incorrectly indicates the presence of a condition, disease, or effect when it is not present (e.g., a diagnostic test indicating a disease when the patient is healthy)
False negative	A result that incorrectly indicates the absence of a condition, disease, or effect when it is present (e.g., a diagnostic test failing to detect a disease in a patient who has it)
Ground truth	The objective, accurate, and verified information or data used as a reference standard to evaluate the performance of a model, test, or algorithm (e.g., biopsy results used to validate a diagnostic imaging tool)
Bias	Systematic errors that cause the model’s predictions to consistently deviate from the true values or outcomes, whether because of flaws in data, assumptions, or the design of the model
Fairness	The ethical principle to ensure that models do not produce biased or discriminatory outcomes, particularly against specific groups or populations
Calibration	The degree to which the predicted probabilities of an outcome match the actual observations. A well-calibrated model means that if it predicts a 30% risk of an event (e.g., a patient developing a disease), the event should occur approximately 30% of the time in reality
Equalized odds	A fairness criterion in machine learning where a model's predictions are considered fair if the true positive rates and false positive rates are equal across different subgroups (e.g., different demographic groups)
Equality of opportunity	A fairness metric that requires a model to have equal true positive rates (or sensitivity) across different subgroups
Equalized error rates	A fairness criterion that requires a model to have equal error rates (e.g., false positive and false negative rates) across different subgroups
Predictive rate parity	A fairness condition where the positive predictive values and negative predictive values are equal across different subgroups
Equal calibration	A fairness criterion where the predicted probabilities of an outcome are well-calibrated and equally reliable across different subgroups. This means that if a model predicts a 30% risk of an event occurring, the event should occur approximately 30% of the time within all groups

## Main text

### What do we mean by “algorithms”?

**Fig. 1:**
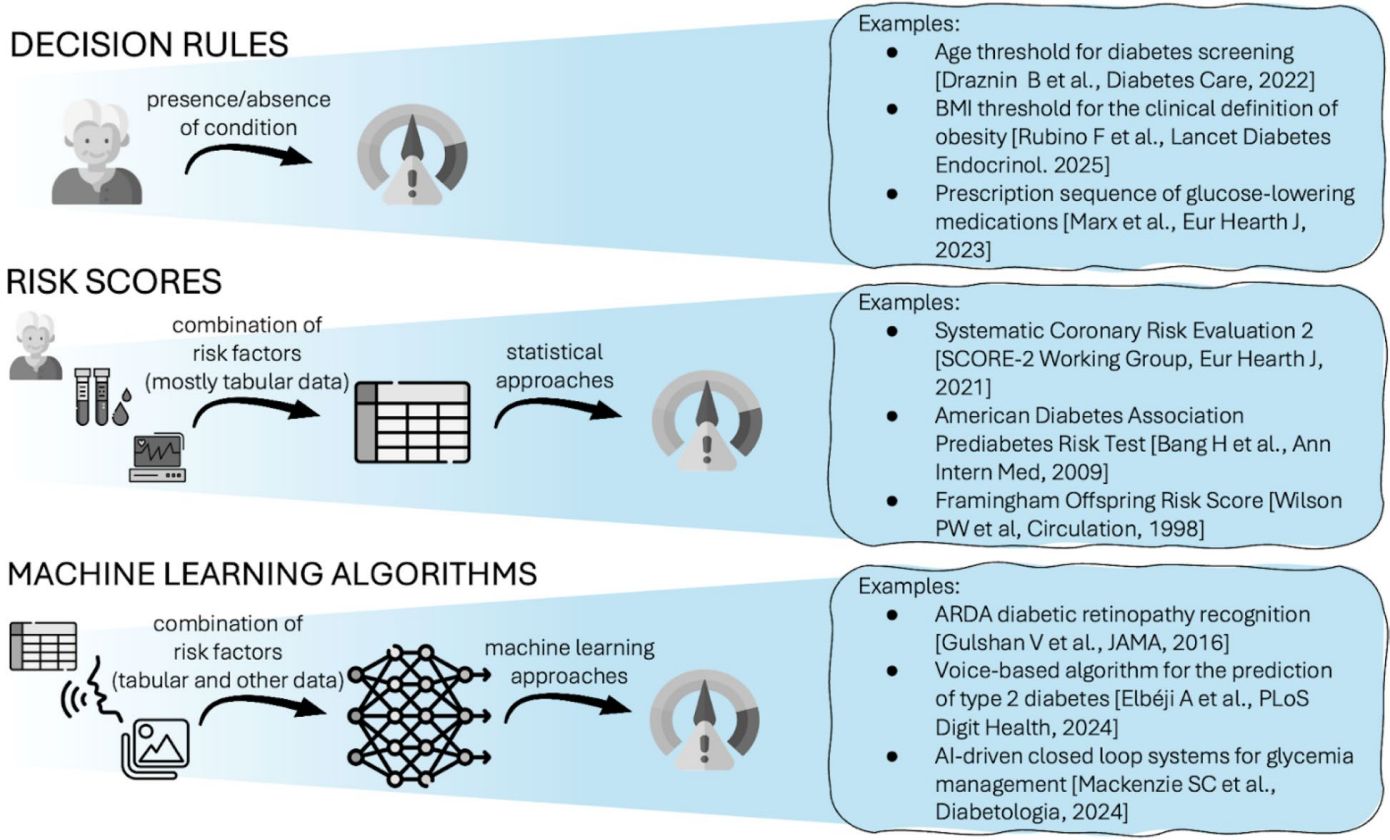
Broad definitions of healthcare algorithms

In this paper, we broadly define ‘algorithms’ as any automated rules or tools that (aim to) assist in streamlining and supporting clinical decision-making processes. These tools range from the most straightforward rule-based systems, like age thresholds for screening, to AI models that analyze medical images and predict patient outcomes (Fig. 1). We choose this broad definition since any algorithm, regardless of complexity, can be subject to biases that may disproportionately impact certain patient groups.

For example, thresholds for initiating cancer screening, such as starting mammography at age 40, are rule-based systems designed to guide clinicians. However, these thresholds can inadvertently reinforce biases if they fail to account for population differences. In the USA, research has shown that to achieve similar detection rates of breast cancer, mammography screening should begin 10 years earlier for Black patients compared to White patients, highlighting the need for tailored, equitable guidelines [7]. Similarly, the clinical definition of obesity has historically relied on BMI thresholds. Still, BMI’s effectiveness as a diagnostic tool varies significantly across age, sex, and ethnic groups due to differences in body composition and health risks [8]. Another example of a simple rule-based algorithm is the 2023 ESC Guidelines for managing cardiovascular disease in patients with diabetes, where medications for lowering cardiometabolic risk are recommended based on the presence or absence of early symptoms related to various diabetic complications [9]. Despite known sociodemographic and metabolic differences across ethnicities, whether this algorithm delivers equitable care remains unclear.

Risk scores, which integrate multiple factors to estimate the likelihood of a specific outcome, constitute slightly more complex tools for clinical diagnosis and prediction. For example, the primary aim of the Systematic Coronary Risk Evaluation 2 (SCORE2) model is to accurately predict an individual’s 10-year risk of CVD. SCORE2 provides tailored risk estimates by stratifying the European population into four regions based on average national CVD risk levels [10] and stratified by sex [11]. Another example is the Steno Type 1 Risk Engine (ST1RE), developed for individuals with type 1 diabetes who face unique cardiovascular risks [12]. General models designed for the broader population or those with type 2 diabetes were found to underestimate CVD risk among individuals with type 1 diabetes, highlighting the need for such tailored algorithms.

AI models are at the forefront of algorithmic complexity. Google’s Automated Retinal Disease Assessment (ARDA) was among the first AI tools to accurately analyze retinal scans for the early detection of diabetic retinopathy, a complication of diabetes that can lead to blindness [13]. Since then, hundreds of AI-based models have been developed for similar purposes, using advanced machine learning to detect diseases and predict outcomes [14]. However, as AI models increasingly inform clinical decision-making and resource allocation, concerns about bias and fairness naturally arise. These models may systematically underperform for certain populations due to biased training data, structural inequities, and unequal access to care, potentially leading to missed diagnoses or unequal treatment recommendations that disproportionately affect already vulnerable groups. Additional challenges include explainability, as healthcare professionals often need to identify actionable risk factors [15]. For instance, state-of-the-art dermatology AI models have shown substantial limitations when tested on more diverse skin biopsy datasets, because they were trained on datasets containing predominantly white populations [16]. While AI-specific issues are important, our focus is on challenges that span across all algorithmic models, not solely those unique to AI.

### Trickle-down bias

SCORE2 is a healthcare algorithm implemented in Europe designed to predict individuals’ 10-year risk of cardiovascular disease. In the development phase of SCORE2, researchers categorized Europe into four cardiovascular risk regions and calibrated the algorithm based on the average risk levels within each region [10]. However, recent studies from Europe showed differential performance of SCORE2 between sexes and age groups [17, 18], and analyses from the Netherlands revealed that SCORE2 underperforms for individuals of low socioeconomic status and those of non-Dutch origin, recommending the importance of supplementing SCORE2 predictions with clinical judgment when assessing risk in these population groups [19, 20]. A key driver of such disparities lies in the various sources of bias that influence algorithm development, including broader societal biases that shape the healthcare landscape, biases embedded in the data used to develop algorithms, and biases introduced during algorithmic design. All of the above may result in biased predictions when applied to marginalized groups.

When an algorithm is developed using a limited dataset that excludes marginalized groups, this can result in biased predictions. An example of such a *data bias* has been observed in the Framingham Heart Study cardiovascular risk scoring algorithm, which performed well in predicting outcomes for white populations of European descent but overestimated the average risk of coronary disease when tested in a more representative sample of British men [21]. Similar performance disparities have been observed with the Framingham Offspring Risk Score, a prognostic algorithm for predicting type 2 diabetes, which systematically overestimated risk for non-Hispanic Whites while underestimating risk for non-Hispanic Blacks [22]. Notably, the Framingham Heart Study and the Framingham Offspring Risk Score were developed using datasets comprising an overrepresentation of White participants. However, challenges such as non-random missing data, differential measurement error, or issues during data collection can still arise even in a complex dataset with diverse and well-represented populations. The observed over- and under-predictions in the Framingham algorithms can have profound implications for patient care, influencing the allocation of healthcare resources, for instance, administration of medications, prioritization of preventive actions, or allocation of hospital beds—all of which have the potential to exacerbate healthcare inequities.

A lack of diverse data is not the sole driver of unfair algorithms. Biases can arise when specific data characteristics are ignored in algorithmic design. For example, a deployed healthcare algorithm in the USA prioritizing people for preventive programs demonstrated *algorithmic bias* by using healthcare costs as a proxy for health needs. This approach falsely suggested that Black patients were healthier than White patients with equivalent comorbidities and conditions due to factors such as differential access to healthcare [3]. As a result, the algorithm identified fewer Black patients as needing additional care, perpetuating already existing disparities in access and care between population strata. However, revising the algorithm to eliminate cost-based assumptions successfully addressed this disparity, highlighting the importance of critically assessing proxies and assumptions used in algorithmic design.

Explicit algorithmic consideration of sociodemographic and socioeconomic drivers of disease is key to addressing algorithmic biases [2, 22]. However, explicitly considering race or ethnicity as a factor is debated and can itself introduce algorithmic bias and contribute to further racial disparities in decision-making and resource allocation [23], as demonstrated in the Vaginal Birth after Cesarean Delivery (VBAC) algorithm. Introduced in 2007, the VBAC algorithm was designed to assess the safety of vaginal delivery after a previous C-section by evaluating factors such as maternal age, the reason for the prior C-section, and the time elapsed since it occurred [24]. However, the algorithm also included race and ethnicity, predicting a lower likelihood of success for individuals identified as African American or Hispanic. While cesarean deliveries are sometimes necessary, vaginal deliveries are associated with significant benefits, including lower surgical risks, faster recovery times, and fewer complications in subsequent pregnancies. Reliance on the VBAC algorithm led to non-White women being disproportionately steered toward cesarean deliveries; the underlying reasons for these disparities revealed a deeper issue: The original VBAC algorithm predicted worse outcomes for non-White women, likely due to socioeconomic factors such as income, education level, and insurance type [25]. This example highlights the importance of addressing how *social biases, racism, and structural inequities* (e.g., healthcare literacy, access to care, socioeconomic status) influence health outcomes. If these factors are causal connections to disparities, failing to identify and tackle them perpetuates harm. By embedding these biases in algorithms, they become hidden and legitimized rather than being uncovered and resolved [25]. Recognizing these issues, the researchers of the original VBAC algorithm revised it in 2021, removing race from the algorithm [26].

**Fig. 2:**
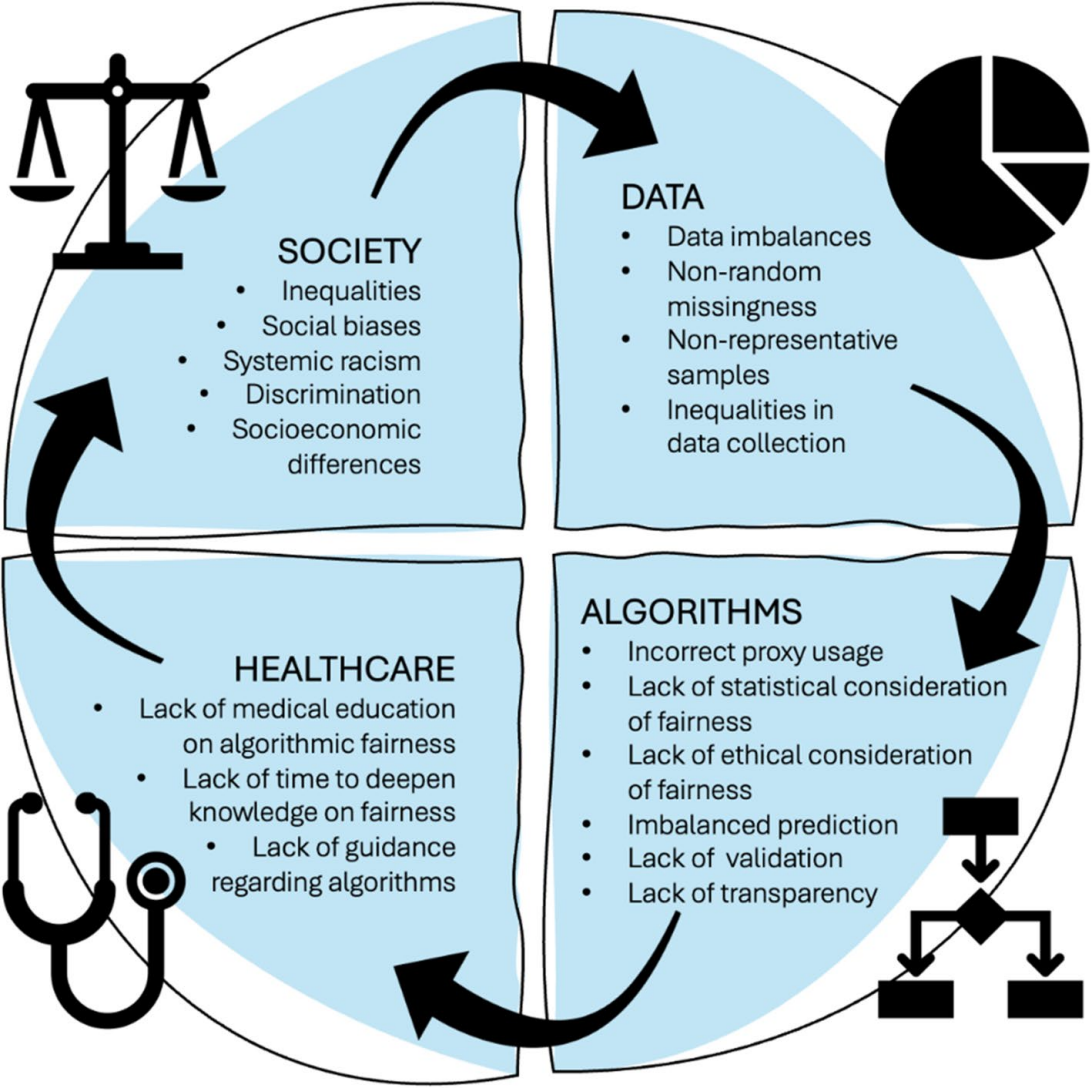
The vicious cycle of bias across society, data, algorithms, and healthcare

Social, data, and algorithmic biases do not exist in isolation. When algorithms are developed and deployed without careful consideration of underlying biases and regular updates to address emergent issues and constantly evolving landscapes of health and disease, there is a risk of creating reinforcing feedback loops: *biases from algorithm-aided decisions themselves can trickle down to healthcare and societal biases*. This feedback loop can sustain or amplify existing inequalities [27] (Fig. 2). Using insights from our clinician and patient coauthors, we have developed a set of questions for healthcare professionals to query algorithms used in their practice (Table 2). Our goal with these questions is to encourage healthcare professionals to assess algorithms critically, consider counterfactual scenarios (“what if” kind of questions), challenge existing practices, and stimulate discussions among colleagues about the legitimacy of algorithmic decision-making in specific clinical situations.

**Table 2:** Critical reflection points on algorithms from a fairness perspective

Bias category	Guiding question	Examples of specific considerations	Use case: Pediatric gastroenterology^a^	Use case: Fetal medicine^b^
Societal biases	Who was involved in the development of this algorithm?	• Was the algorithm developed by researchers only or including clinicians, patients, and other stakeholders? • Do the developers reflect a diverse range of experiences and perspectives?	The algorithms used for Crohn’s Disease (CD) [28] and Ulcerative Colitis (UC) [29] disease activity tracking were developed primarily by clinician researchers (mixed affiliations), without the involvement of patients or other stakeholders. The developers primarily come from a narrow field. It would be important to include patients in the design, with many journals now requiring this	The fetal growth charts [30–32] were developed by clinician researchers within obstetrics, pediatrics, epidemiology, biostatistics, and mathematics with expertise in cohort studies and public health. No patients or other stakeholders were involved in the process
	What is the goal of the algorithm?	• Has the algorithm been developed in response to an outspoken need from the patient community? • If so, were historically marginalized groups’ voices represented?	The algorithm was not developed in response to an outspoken need from the patient community. In fact, patient priorities are often overlooked in algorithmic design and use [33]. For example, fatigue and quality of life are clear patient priorities; even in the “mild” category, patients often experience fatigue. This aspect is often ignored by clinicians	The algorithm was not developed in response to an outspoken need from the patient community
	What is the target population of the algorithm?	• Can the algorithm be applied broadly in a population? • Are there any assumptions behind this algorithm that might disproportionately affect historically marginalized groups?	The algorithm is broadly applied in pediatric inflammatory bowel disease (IBD) care worldwide, but with important limitations. A key assumption behind this algorithm is that it can be universally applied in any pediatric population	The algorithm is broadly applied in the Danish population. Scandinavian data is used as the baseline for normal fetal weight estimation [32]. Thus, Scandinavian data serve as the reference standard for fetal weight calculations, assuming that these calculations can be applied to all mothers in Denmark
	What are the potential consequences of adopting this algorithm in healthcare?	• How might this algorithm perpetuate societal power imbalances, systemic biases, and existing disparities in healthcare access or outcomes in the short- and long-term? • How might this algorithm affect resource allocation, and could it lead to prioritization that harms certain groups?	The algorithm might perpetuate imbalances and affect resource allocation through multiple paths. For example, underreporting among families with low health literacy (correlated with low socioeconomic status) leads to lower scores, which may delay diagnosis or treatment. Subjectivity in moderate scores may unintentionally reflect implicit bias. There is a higher chance of complications in low-resource settings, with lower probabilities of access to advanced therapy. Even in Denmark, disparities exist, and, for instance, those who live farther from a major hospital might receive delayed treatment, which can impact the disease course in CD	Using population-specific reference curves is crucial because ethnic minorities with naturally smaller (e.g., Southeast Asian descent) or larger (e.g., Chinese descent) fetuses may be misclassified as small (SGA) or large for gestational age (LGA) despite normal development. Since Denmark’s fetal growth standards are based on Scandinavian populations, this misclassification can lead to unnecessary medical surveillance, increased testing, and potential stress for parents, while also straining healthcare resources. Additionally, these errors may impact clinical decisions around monitoring, delivery timing, and risk stratification for neonatal complications
	Are there potential risks that this algorithm could inadvertently create?	• Does the algorithm perpetuate inequalities by benefitting marginalized groups less as compared to others? • Does this algorithm create barriers to care for marginalized populations?	Yes, there is a high risk for this. For example, language, health literacy, or cultural gaps may hinder symptom interpretation; with current time constraints, clinicians might not be able to devote enough time to explaining what frequent defecation means or clinicians might assume that everyone is informed, e.g., in relation to how to detect blood in stool, what soft stool means, which might not be the case for those with lower health literacy. On the other hand, high-literacy families may overreport symptoms to inflate scores and secure better care	Southeast Asian women are more likely to develop gestational diabetes mellitus (GDM), but fetal size norms based on Scandinavian charts don’t reflect their typically smaller fetal growth patterns [34]. Having a LGA fetus, being an established risk factor, normally flags mothers for referral for GDM screening. However, larger fetuses in Southeast Asian women may be misclassified as normal, causing clinicians to miss or delay follow-up for GDM, despite higher risk. This misalignment leads to fewer referrals and delayed diagnosis, perpetuating disparities in maternal and fetal health, both short- and long-term, since GDM increases the risk of metabolic diseases for both mother and child
Data biases	How representative is the data used to build this algorithm?	• Is there an imbalance in the dataset in terms of the representation of marginalized groups? • Are marginalized groups well represented in the data, proportional to the populations? • What is the magnitude of the data imbalance?	Yes, there is a significant imbalance in the dataset with respect to the representation of marginalized groups. The algorithm was developed in a mostly white population, as these diseases emerged with high prevalence in Northern European and Canadian populations. Lots of novel research emerges from homogeneous, mostly white samples	Yes, there is an imbalance in the dataset with respect to the representation of marginalized groups. The algorithm may overlook clinically meaningful ethnic and regional variation in fetal size, particularly in abdominal circumference and estimated fetal weight, both key predictors of perinatal risk
	Are there any biases in the data collection process?	• Is missingness correlated with sensitive attributes such as race, ethnicity, sex, gender, sexuality, age, and disability status? • Have data biases been critically evaluated, quantified, and addressed?	Ethnic minorities, immigrant populations, families with low socioeconomic status, children from underserved communities, and patients from developing countries are largely lacking from the model development phase. Data biases have not been evaluated	Ethnic minorities and immigrant populations are underrepresented in the development of the algorithm
	What are the sources and types of data used?	• Do the data sources reflect the diversity of the populations the algorithm is meant to serve? • Does the real-life environment where the algorithm is meant to be adopted match the quality of the data used for the algorithm?	The prevalences of CD and UC are now rising in developing countries. However, the algorithm has not been updated in response to this phenomenon. The same algorithm is applied between ages 0–18, which might not be appropriate. Pediatric IBD care sometimes relies on research undertaken in adult populations (e.g., related to some advanced therapies)	The Verburg and Hadlock formulae, along with the associated growth curves, were all derived from predominantly white populations. These growth standards have not been updated to reflect the growing diversity of Denmark’s population, including an increasing proportion of immigrants
	What is the provenance of data?	• Have legal, ethical, and privacy guidelines been followed during data processing? • If the data comes from marginalized populations with a history of exploitation, what safeguards are in place to ensure ethical use and prevent harm?	These were likely followed. As the algorithm was developed in a predominantly white population, the second question here is not applicable	These were likely followed. As the algorithm was developed in a predominantly white population, the second question here is not applicable
Algorithmic biases	Is fairness an explicit consideration in the development of the algorithm?	• How is fairness defined in the development of this algorithm? • What fairness metrics were used during development?	Fairness was not defined and evaluated	Fairness was not defined and evaluated
	What steps were taken to identify and mitigate potential biases during the algorithm’s development?	• Have the developers attempted to correct data imbalances? • How does the algorithm handle data missingness? • Have the developers attempted to place fairness constraints on algorithm development or otherwise tune algorithms for fairness?	Fairness was not defined and evaluated	Fairness was not defined and evaluated
	How are variables and outcomes defined?	• Does the algorithm use predictors or outcomes that serve as proxies for sensitive attributes and/or social determinants of health, such as income, insurance type, or access to healthcare?	Such features were not included in this model, which tracks symptoms and disease activity over the disease course	Such features were not incorporated into the model, which assists clinicians in assessing fetal growth based on standardized growth charts
	Has the algorithm been tested in different scenarios?	• Does the algorithm provide equally stable individual prediction for underrepresented groups as for the majority group? • Has the algorithm been evaluated using external datasets in various contexts? • Do the developers of the algorithm adhere to a development checklist such as TRIPOD + AI?	This was not evaluated	This was not evaluated
	Has a management plan been put in place for the algorithm?	• How do the algorithm’s maintainers account for emerging societal trends? • Is there a plan for updating the original dataset with changes in healthcare practices?	The algorithm has not been updated in response to, for example, rising prevalences of CD and UC in the Global South. No knowledge of plans in this realm	The algorithm has not been updated to reflect demographic changes, such as the growing proportion of the immigrant population in Denmark. Despite clinicians recognizing ethnic differences in fetal growth, the prevailing approach is to “treat all mothers equally” without explicitly accounting for ethnicity, which can be counterproductive
	How transparent is the algorithm, and can clinicians and patients easily understand how decisions are made?	• If a ‘black-box’ model is being used, have sufficient efforts been made to enhance its explainability and interpretability? • Can the causal drivers of disease be identified to enable effective intervention? • Have healthcare professionals received adequate training on the algorithm’s use?	This is not a'black-box'AI model, but a scoring algorithm composed of predefined features and thresholds, which is interpretable and actionable for clinicians. Causal drivers can be intervened upon. There is no formal training; clinicians learn the use of this algorithm via daily practice, with some important aspects emerging via continued use. For example, defecation during the night can inflate scores, but there are no questions regarding when the patient had dinner, which is a determinant of night defecation (i.e., night defecation is a serious symptom if the patient had an early dinner but could be normal with a very late dinner). Clinicians interpret the scores subjectively and decide the best course of action	This is not a'black-box'AI model; rather, the process of comparing fetal size with an established growth curve is transparent and interpretable, following a clear, standardized flowchart that guides clinical decision-making. Nuances in terms of how to handle ethnic differences is learned through clinical practice
Overall reflections			Clinicians apply algorithms without much critical reflection on provenance, fairness, disparate impact, or potential biases. Reading this paper and going through the questions were very useful and inspirational in becoming more reflective about algorithmic limitations and broader implications, including concerns about fairness, subjectivity, and transparency. There would be great value in institutionalizing discussions through journal clubs to critically engage with the growing number of algorithmic tools	The national guidelines rely exclusively on Scandinavian fetal weight references, overlooking ethnic variation. As a result, women carrying naturally smaller babies may be classified as having SGA fetuses and subjected to additional monitoring and anxiety, despite clinicians accepting this trade-off to reduce the risk of perinatal death. However, clinicians should not be handed guidelines and charts without also being educated on their limitations; they must understand these shortcomings to provide individualized care and effectively explain the rationale behind the formulae and algorithms informing their clinical decisions

^a^Brief description of the Pediatric Gastroenterology use case: This use case involves a clinical scoring algorithm used in most pediatric inflammatory bowel disease (IBD) centers to assess disease activity in pediatric patients with IBD, specifically Crohn’s Disease [28] and Ulcerative Colitis [29]. The tool stratifies patients into “mild,” “moderate,” or “severe” risk categories based on a range of non-invasive clinical inputs, including symptom reports and, in some cases, laboratory tests. These scores help track disease activity and progression, guiding decisions about treatment escalation, particularly the use of advanced therapies. In case of a “mild” score, watchful waiting is recommended, with a follow-up in 3–6 months. In case of a “severe” score, a patient visit is recommended with an in-person diagnostic process, with additional blood tests, and potentially endoscopy. Highly severe scores warrant immediate treatment with steroids. “Moderate” scores require further consideration, with clinicians analyzing what elevated the scores and usually also investigating an additional fecal marker; additional in-person patient visits could be warranted if the patient is at the higher end of a moderate score. These disease activity scores are widely implemented in practice, with the interviewed pediatrician evaluating approximately ten patients per day

^b^In Denmark, fetal development is assessed using standardized models throughout pregnancy: the Verburg model [31] is used nationally in the first trimester to estimate gestational age based on crown-rump length, while the Hadlock formula [30], based on ultrasound biometry measurements like abdominal and head circumference and femur length, is used in the second and third trimesters to estimate fetal weight. These tools help detect growth abnormalities such as intrauterine growth restriction and macrosomia, guiding clinical interventions. Since 1997, Denmark has used Scandinavian population data [32] as the reference standard for fetal weight estimation (growth curves) to ensure local relevance. These assessments are part of routine pregnancy care, with additional fetal scans recommended when there are signs of growth concerns or reduced fetal movement

### How to navigate algorithmic fairness metrics?

Before exploring fairness metrics, it is important to define fairness in the context of healthcare algorithms. Fairness refers to the principle that these algorithms should deliver equitable outcomes across all patient groups, regardless of race, gender, socioeconomic status, or other demographic factors. This involves identifying and addressing biases in training data and ensuring that predictions do not systematically disadvantage any group. In practice, fairness means building algorithms that are both accurate and equitable, with reliable performance across diverse populations. It also requires transparency in model development, monitoring, and evaluation so that disparities can be recognized and addressed. The ultimate aim is to support healthcare decisions that are inclusive and just, rather than reinforcing existing inequalities.

According to recent guidelines on developing clinical prediction models, such as the TRIPOD+AI, newly developed diagnostic or prognostic prediction models should address fairness during development [5]. While adherence to these reporting guidelines has historically been very poor [35], algorithmic fairness considerations have gained traction in recent years, and algorithmic fairness metrics and toolkits have been under development [see curated list of tools in [36]. Thus, healthcare professionals can expect to encounter scientific literature on the fairness and bias of historic or newly developed algorithms. In the context of diagnostic and prognostic algorithms, researchers generally compare ground truth labels (whether an individual has the disease or has developed the disease) and the algorithm’s prediction on whether the individual has or will develop the disease. In this context, we will introduce two key groups of algorithmic fairness metrics related to prediction parity (equality) across two or more population groups of interest.

**Fig. 3:**
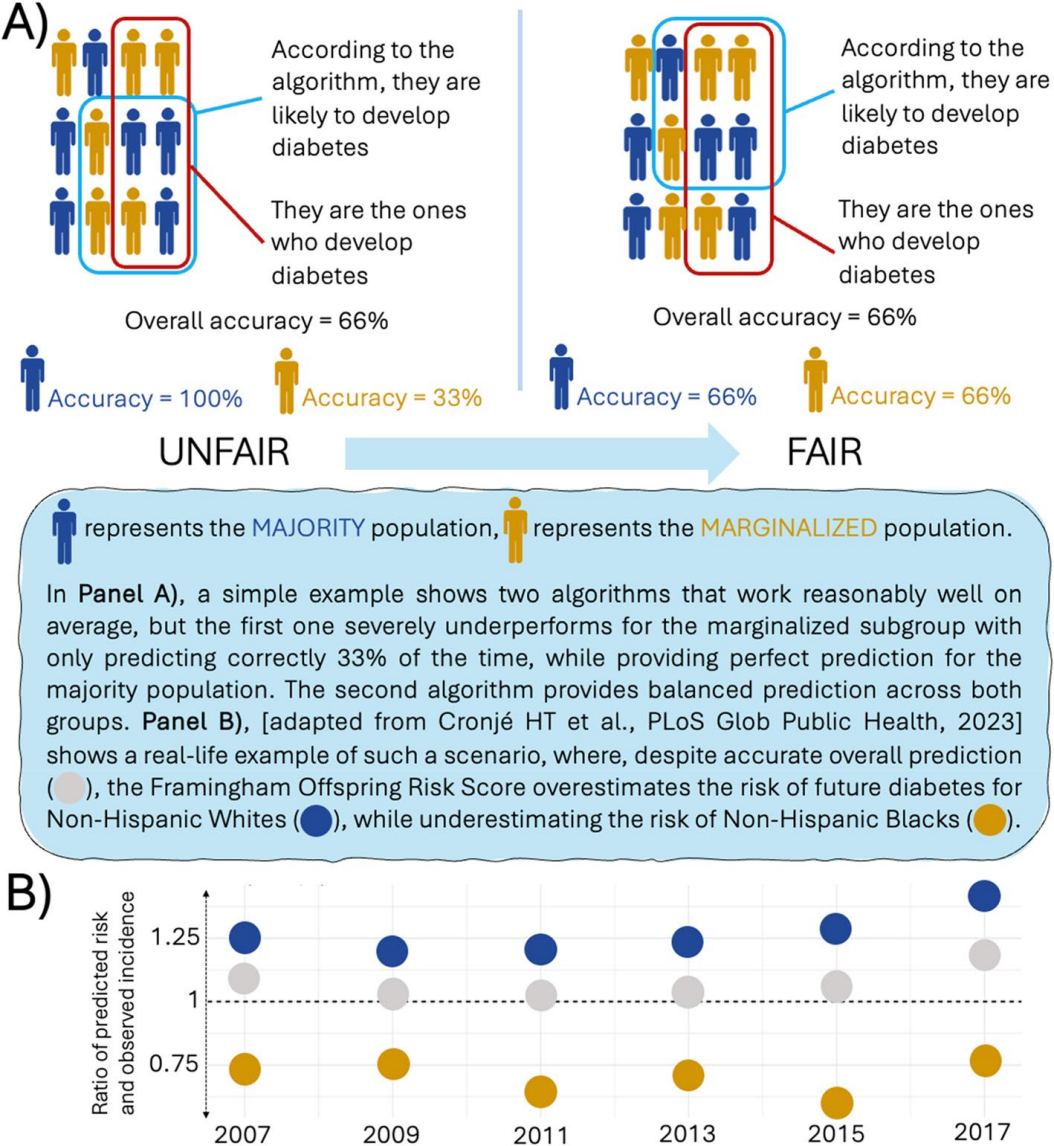
The logic of algorithmic fairness metrics and a real-life example of disparities in prediction errors across groups

The first group of fairness metrics looks at error rates, such as false positive rates, false negative rates, false discovery rates, and false omission rates, and evaluates whether these types of errors are equally distributed across different groups [37]. For example, in a diabetes prediction algorithm, a false positive means the model wrongly predicts that someone will develop diabetes. This creates “false alarms,” leading to unnecessary use of healthcare resources and possibly “alert fatigue”—a phenomenon where many false alarms inadvertently cause clinicians to ignore real warning signs [38]. On the other hand, a false negative means the model predicts someone will stay healthy while they actually will develop diabetes: these are “missed cases” where people do not receive preventive care. If error rates are imbalanced across racial groups, an algorithm could unfairly underprioritize or over-allocate resources like tests, scans, and treatments for specific population subgroups (Fig. 3). Using false discovery rates and false omission rates as fairness metrics might be particularly appealing when an optimistic prediction involves significant costs, such as when diagnosing a disease requires a substantial allocation of healthcare resources [39].

The second group of fairness metrics is concerned with calibration, or how well predicted risks reflect actual outcomes across groups. While both groups of metrics rely on actual outcomes, calibration assesses whether the predicted probabilities correspond with the observed frequencies of the event. For example, if a model predicts a 20% risk of diabetes, then ideally, 20 out of 100 people with that prediction should actually develop the disease. Calibration fairness requires that this reliability holds equally across all demographics. An algorithm may be considered unfair if it systematically overestimates risk in one group while underestimating it in another. We observed a real-life example of unequal (thus, unfair) calibration in the performance of the Framingham Offspring Risk Score to predict type 2 diabetes, which was more likely to underestimate the risk in Black individuals and overestimate it in White individuals [22].

In scientific publications, key terms for the first group of fairness metrics include “equalized odds,” “equality of opportunity,” and “equalized error rates.” In contrast, terms like “predictive rate parity” and “equal calibration” are commonly associated with the second group. However, these terms are sometimes used imprecisely, confusing the literature [40–42]. We encourage readers to focus less on the specific terminology and instead prioritize understanding which aspects of the algorithm are being statistically compared across population groups. When access to sensitive attributes is limited, algorithm developers must turn to fairness techniques based on the setting. One such approach is *individual fairness*, which posits that similar individuals should receive similar treatment [43]. Rather than relying on protected-group membership, this principle uses task-specific similarity metrics, based on selected features, to ensure consistent algorithmic decisions across individuals who are deemed “similar.” While individual fairness avoids the need for sensitive attributes, its effectiveness depends heavily on how well these similarity metrics reflect meaningful and equitable comparisons.

Fairness metrics often contradict each other, and satisfying one metric does not guarantee fairness across others—this phenomenon is usually referred to as the *impossibility theorem*. Fairness is inherently subjective, and certain scenarios may call for specific metrics; we recommend looking for explicit explanations for why a particular metric was chosen in algorithmic development or evaluation. As another consideration, achieving fairness sometimes requires sacrificing overall predictive performance. While recent research explored ways to overcome this trade-off [44], there is still an ethical dilemma: Is it justifiable to reduce performance for previously advantaged groups, potentially causing harm, to improve fairness and outcomes for marginalized or disadvantaged groups? Therefore, we recommend exploring scientific literature that examines statistical fairness metrics and addresses healthcare algorithms’ underlying ethical and societal implications [39, 45, 46].

## Conclusions

Beyond statistical fairness metrics, it is crucial to consider the algorithms’ ethical and societal implications concerning patient needs and preferences before utilizing such tools in clinical practice. Regulatory and ethical constraints on collecting sensitive attributes significantly influence which fairness analyses and metrics are feasible. In the USA, race and ethnicity are routinely captured in clinical and administrative datasets, often driven by federal reporting requirements and Office of Management and Budget standards. In contrast, countries like Denmark, guided by the GDPR and the Danish Data Protection Act, treat race and ethnicity as special categories of personal data. Their collection is prohibited or tightly restricted unless under narrow, legally defined circumstances. Consequently, understanding and addressing fairness in healthcare must be grounded in the specific legal, ethical, and cultural contexts of each country, recognizing that the methodologies effective in one setting may not be transferable in another.

The interviewed clinicians recognized that most algorithms were developed without input from patients, especially from underrepresented populations, and patients were not informed about how these models are developed during the treatment phase; therefore, they emphasized the importance of including patient voices. Through our interviews with patients with lived experience of a chronic disease, it became clear that patients want to understand their risks for future diseases and feel empowered to take proactive steps toward preventing or managing disease complications and comorbidities. While some patients value detailed information about their risk and probability of developing future diseases, others may find such information overwhelming, confusing, or frightening. Instead of debating whether risk must be communicated or not, we emphasize the importance of *building trust* between healthcare professionals and patients. When trust is established, patients and providers can engage in open, informed decision-making, leading to better adherence to recommendations and improved outcomes. To foster this trust, it is important to communicate transparently with all advantaged and marginalized patients about whether healthcare processes are more or less beneficial to them than others and whether any actions can be taken to address these disparities. Without trust, overburdened healthcare systems face a significant risk of alienating patients. From the clinicians we interviewed, we learned that some healthcare professionals can often only afford to spend 10–20 min with each patient, including administrative tasks, which could result in patients feeling unsupported or unheard. This also means that clinicians may not have the time to explain how risk prediction algorithms function. This was also corroborated by the patients we interviewed. As a result, patients may increasingly turn to other sources for support, advice, and information, such as social media, specialized websites, chatbots, television, peer networks, or alternative digital health tools [47, 48]. However, access to these resources and healthcare literacy vary widely across demographic and socioeconomic groups, introducing another route to perpetuating inequalities.

Our specific points for critical reflection for healthcare professionals regarding algorithms are summarized in Table 2. First, we recommend *reflecting on algorithms* by critically evaluating them from both methodological and ethical perspectives, and second, we recommend *fostering inclusive cultures* where fairness and bias in algorithms are openly discussed with peers, patients, and interdisciplinary research teams. We also advocate for *systematic education on algorithmic fairness* in clinical care. Those developing and implementing these models are responsible for introducing them transparently [49] to healthcare professionals. Clear explanations of how these models function and where biases may arise give clinicians a fair opportunity to identify concerns. While existing frameworks are often aimed towards healthcare regulators, health technology manufacturers, and developers of medical algorithms [39, 40, 49], those who interact with these algorithms in care settings may have limited influence over how they are designed or implemented, as we also noticed from our discussions with patients and healthcare professionals. It is therefore our intention that these critical points for reflection will support clinicians and healthcare professionals in their attempts to make meaningful, fair, and unbiased decisions. We encourage healthcare professionals to engage in thinking about counterfactual scenarios, asking difficult “what if” and “all else being equal” questions related to clinical algorithms. This is directly in line with the *counterfactual fairness* approach, a methodology that uses causal reasoning to assess whether an algorithm’s prediction for an individual would remain unchanged if their sensitive attribute were altered in a hypothetical scenario, holding all else constant [50, 51]. We encourage healthcare professionals to initiate internal discussions on fairness, such as hosting journal clubs, inviting experts and algorithm developers to educate smaller groups, and integrating algorithmic fairness into conferences of medical societies. By making this information more accessible and proactively addressing bias, we can better support equitable patient care. The interview process prompted self-reflection among clinicians, and we experienced strong support for institutionalizing such critical discussions to promote algorithmic literacy and thoughtful application.

The core principle of personalized/precision medicine is to accurately identify individuals or groups with specific needs and address those needs in a targeted way. In societies marked by systemic racism, discrimination, and health inequalities, it is of utmost importance to recognize that individual needs will differ. Simply offering patients the same care risks maintaining or exacerbating these inequalities. As AI-based prediction tools become increasingly embedded in clinical practice, their potential to mitigate or amplify health disparities will grow. Without proactive efforts to ensure fairness, inaction could lead to healthcare systems perpetuating societal disparities, leaving medical care a privilege for those who already benefit the most [52, 53].

## Supplementary Information

Supplementary Material 1.

## Data Availability

No datasets were generated or analysed during the current study.

## Abbreviations

AI: Artificial intelligence
ARDA: Automated Retinal Disease Assessment
CD: Crohn’s disease
CVD: Cardiovascular disease
GDM: Gestational diabetes mellitus
IBD: Inflammatory bowel disease
LGA: Large for gestational age
SCORE2: Systematic Coronary Risk Evaluation 2
SGA: Small for gestational age
ST1RE: Steno Type 1 Risk Engine
UC: Ulcerative colitis
VBAC: Vaginal Birth after Cesarean Delivery
