# Supplementary material for: Navigating fairness aspects of clinical prediction models
Source: BMC Med. 2025 Oct 17;23:567. doi: 10.1186/s12916-025-04340-3 (PMC12535043; doi:10.1186/s12916-025-04340-3)
Supplement: Supplementary file 1 — Supplementary Material 1. [file 12916_2025_4340_MOESM1_ESM.pdf]

Text S1. Interview protocol and questions for healthcare professionals and patients with lived experience of type 2 diabetes

1. **Protocol**

Two of the co-authors (KC and TVV) conducted five semi-structured qualitative interviews via secure video call between January 7 and 10, 2025. The interviewees included two clinicians (SBB and OLD) and three patients with lived experience of type 2 diabetes (HG, SKL, and HH). All interviews were conducted in English, which was not the participants' first language. As part of our participatory research design, we offered all informants the opportunity to be included as co-authors.

Both clinicians were identified through members of the core research team (TVV, TLN, and AH), who had prior personal connections with them. We contacted the clinicians via email with information about the project, the participatory design, and an invitation to participate in a 60-minute interview. Both agreed to participate.

To recruit patients with lived experience of type 2 diabetes, TVV posted announcements in the Facebook groups "Diabetes 2 - Danmark," "Vi med Diabetes Typ 2," and "Diabetes type 1." Of the five individuals who expressed interest, TVV contacted each via Messenger with further information about the study, the participatory framework, and an invitation for a 60-minute interview. Three agreed to participate.

KC and TVV developed the interview guide. The participatory research approach was explained in detail to all clinician participants, who had prior experience with the biomedical publishing process. For patients, we carefully explained what it means to publish an academic article, the ICMJE criteria for co-authorship, and the nature of our participatory design. After the interviews, the core research team drafted the manuscript and invited all contributors to review and provide feedback. At this stage, HH (a patient) expressed support for the project but preferred to be acknowledged rather than listed as a co-author.

KC and TVV took detailed notes during the interviews, capturing key insights. Once the interviews were complete, they reviewed and synthesized the notes into a list of emerging themes. This list, further refined through input and suggestions from all co-authors, formed the basis for Textbox 2: Critical reflection points on algorithms from a fairness perspective.

In response to reviewer comments during the manuscript submission process, we conducted follow-up structured interviews with SBB and an additional clinician, MM, who we subsequently included as a coauthor. Each interview lasted 60 minutes. In these follow-ups, we used the reflection points in Textbox 2 to walk through two use cases from SBB's and MM's clinical experiences. At the end of the interviews, we asked both clinicians to reflect on their overall experience participating in the project, reviewing the manuscript, and using the Textbox 2 questions as tools for critical reflection.

2. **Announcement and invitation posted in the Facebook groups**

Dear Community!

My name is Tibor, and I'm an Associate Professor at the University of Copenhagen. I'm working on a study about predicting future health issues related to type 2 diabetes, including its complications. My research focuses on how these predictions can be made fairer and more inclusive for everyone.

I'm looking for a few volunteers to help me and become involved in my research. This would involve a short interview or chat (about 30-45 minutes) in English. I'd like to hear from people with different backgrounds, including those who identify as native Danes and those who identify as immigrants or ethnic minorities.

If you're interested or would like to know more, please feel free to contact me directly or leave a comment below and I will reach out. I'd love to hear from you!

Thank you so much,

Tibor

3. **Follow-up message to interested patients via Messenger**

Dear XXXX,

Thank you for your interest in our project! I'd like to share more details so you can decide if you'd like to contribute. Doctors routinely assess patients' personal risk for future diseases using information like age, lifestyle, and blood test results. This process, called "clinical risk prediction," often relies on data from large studies. However, these studies sometimes focus on specific groups (e.g., mostly men or mostly white people), which can make the predictions less accurate for others. We're writing a research paper for healthcare professionals to highlight this issue, explain the related statistics, and suggest ways to communicate these limitations to patients. Here's where you come in: we'd love for you to join us as a co-author! Rather than just sharing your experiences, you'd help shape the paper, ensuring it reflects patients' perspectives. If you're interested, we'd begin with a 1-hour online meeting to discuss the topic. After that, we'd draft the paper using your and others' input, then share it with you for feedback. You'd have a chance to suggest edits, ensure the content is clear, and learn about the publishing process. We'd guide you every step of the way so you feel fully informed about co-authorship. Let me know if you have any questions or concerns, and whether you'd like to participate. If so, I'll send some meeting date options for early January.

Best wishes,

Tibor

4. Questions for patients:

1. Can you walk us through your diabetes journey?
2. Are you familiar with the common risk factors for developing diabetes?
3. Has a healthcare professional ever communicated your personal risk for developing diabetes or related complications?
4. Do you know how personalized risk estimates for diseases like diabetes are calculated?
5. Would you be interested in knowing your personalized risk for a specific disease?
6. If given the choice, would you prefer a clinician to tell you that you have a 5% chance of developing a disease, or simply that there is a chance?
7. Are you aware that some risk factors can differ across demographic groups, such as gender or ethnicity?
8. If a risk prediction model was developed only for men, do you think its results would be relevant for women?
9. How important is it to you that a clinician explains if a healthcare process might work differently for you compared to others?
10. How would you feel if you learned that certain healthcare models were developed using data only from white males or only from females?

5. Questions for clinicians

1. Can you describe your typical daily clinical practice?
2. Are you familiar with the concept of risk prediction or risk scores?
3. How often do you assess patient risk with/without the help of algorithms or tools?
4. Do you discuss your assessments with colleagues?
5. How do you typically communicate risk to patients?
6. Did you learn about risk prediction models during your medical training or later in practice?
7. In your experience, how much trust do clinicians place in risk prediction tools? Are there ever disagreements about the results?
8. Do you know how risk scores are typically developed?
9. Are you aware that some risk scores may perform differently across demographic groups (e.g., sex, ethnicity)?
10. How do you think data imbalances, such as developing an algorithm using only male patients, affect performance for underrepresented groups?
11. Do you know whether [specific algorithm selected for discussion] has been tested across different demographic populations?
12. Do you think patients are meaningfully involved in the decision-making process around their care?
13. In your opinion, is it ethically acceptable to include sensitive attributes like race or ethnicity in prediction models?
14. Do you and your colleagues ever question whether these tools work differently for different people?
15. What is the culture among your colleagues when it comes to educating and sensitizing each other about sensitive issues, such as differences in ethnicity?
